# Causation between Pathway Completion and Reduced Hospital Stay in Patients with Lung Cancer: a Retrospective Cohort Study Using Propensity Score Matching

**DOI:** 10.1007/s10916-020-01570-1

**Published:** 2020-04-21

**Authors:** Hiroki Furuhata, Kenji Araki, Taisuke Ogawa

**Affiliations:** 1 Department of Hospital Institutional Research, University of Miyazaki Hospital, 5200 Kibara Kiyotake-cho, Miyazaki, 8891692 Japan; 2 Graduate School of Medicine and Veterinary Medicine, University of Miyazaki, 5200 Kibara Kiyotake-cho, Miyazaki, 8891692 Japan

**Keywords:** Clinical pathway, Electronic medical record, Lung cancer, Propensity score matching

## Abstract

We have previously demonstrated that clinical pathway completion helps reduce hospital stays. However, our previous results showed only a correlation, not causation. Therefore, the current study’s aim was to analyze the causation between clinical pathway completion and reduced hospital stays for patients with lung cancer. Data were collected from April 2013 to March 2018 from the electronic medical records of the University of Miyazaki Hospital. We used propensity score matching to extract records from 227 patients. Patients were further divided into a pathway completed group and a pathway not completed group; 74 patients in each group were available for data analysis. Our main analysis involved estimating the discharge curve, which was comprised of the in-hospital rate and hospital stay. Additional analyzes were performed to compare the frequency of medical treatments registered in the clinical pathway but not implemented (termed deviated medical treatments). The occurrence of these treatments meant that the clinical pathway was not completed. The main results indicated a decrease in the in-hospital rate of the completion group, compared with the not completed group. The *p* value of the log-rank test was <0.001 for total patients and patients who underwent resection, and 0.017 for patients who did not undergo resection. Additional results indicated that a number of intravenous drips were not implemented, despite their registration on clinical pathways. Our results indicate that clinical pathway completion contributes to improved efficiency and safety. This simplified procedure is expected to be applicable to other diseases and clinical indicators.

## Introduction

### Background

Clinical pathways (CPs) are implemented and continuously revised as part of an overall standard treatment plan to improve medical service. Various research studies have indicated improved clinical indicators (e.g., length of hospital stay, mortality rate, medical cost, and patient satisfaction) by the new implementation or the revision of CPs. Our research group has defined two types of completed CPs. The first involves taking all the medical treatments, including those described on the CP. The second involves taking only the medical treatments described on the CP. In addition, clinical pathway completion rate was extended from a binary system (completed or not completed) to a percentage (0%–100%). Under these definitions, our recent study demonstrated reduced hospital stays and decreased mortality rates (*p* < 0.001) with the occurrence of the first completion [1]. However, only a correlation was shown and a causal relationship between CP completion and clinical indicators could not be shown. Therefore, this study aimed to clarify the causal relationship using propensity score matching (PSM) methods for patients with lung cancer. A number of studies have applied PSM methods to divide patients into targeting groups and control groups, similar to randomized clinical trials. For example, a family history of lung cancer [2], postoperative radiotherapy [3], the use of beta-blockers [4], preoperative radiotherapy [5], intermittent chest tube clamping [6], systematic lymph node dissection and lobe-specific lymph node dissection [7], as well as other variables [8–12], have been used to divide patients into the two groups. Most cancer studies estimate survival curves as related to the *N-years* survival rate as their main outcome. In this study, we focused on hospital stay, a typical short-term quality indicator, in patients that had completed CPs or not. By taking the results of previous studies into consideration, this study suggests the *N-days* in-hospital rate as a measure of the efficiency of the medical service(s) provided. In addition, this study emphasized operations because several guidelines recommend that medical staff consider operations on the associated flowcharts [13–17].

### Objectives

The objectives of this study were to evaluate the effects of CP completion on hospital stay by applying PSM techniques to electronic medical records (EMRs). We hypothesized that patients with CP completion can leave the hospital immediately compared with those without CP completion. CP completion was defined taking all the medical treatment registered on CP. This study was based on the assumption that the length of hospital stay was shorter for patients with CP completion than for patients without CP completion.

## Methods

### Study design and setting

This is a retrospective cohort study using applied PSM techniques in patients with lung cancer. Data were collected from EMRs of the University of Miyazaki Hospital.

Because this study has analyzed EMRs about past hospitalization, there was no recruitment of participants themselves as in typical randomized, intervention, and prospective clinical trials. Moreover, sample size estimation was not operated, because this study has focused on complete survey.

### Data sources

**Fig. 1:**
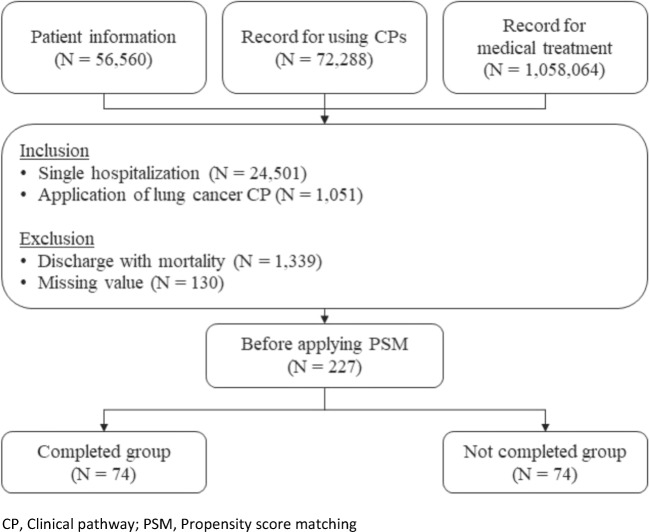
Flowchart with a summary of patient enrollment and propensity score matching

Figure 1 provides a summary of the data preparation and processing used in this study. The cohort for data analysis was created from EMRs from the University of Miyazaki Hospital. The period of data extraction was five years (from April 1, 2013 to March 31, 2018). Three kinds of data were extracted as follows: (1) patient information (e.g., age, sex, date of admission and discharge); (2) record for using CPs (e.g., date of start and end, disease name); (3) record of medical treatment (e.g., type of treatment, date of enforcement). As a summarization the three databases and application of the inclusion and exclusion criteria, 223 patients were extracted for application of PSM. After applying PSM based on sex, age, operation, and complication as explorative variables, two groups (with CP completion and without CP completion groups) were created for data analysis (each 74 patients).

### Variables

**Fig. 2:**
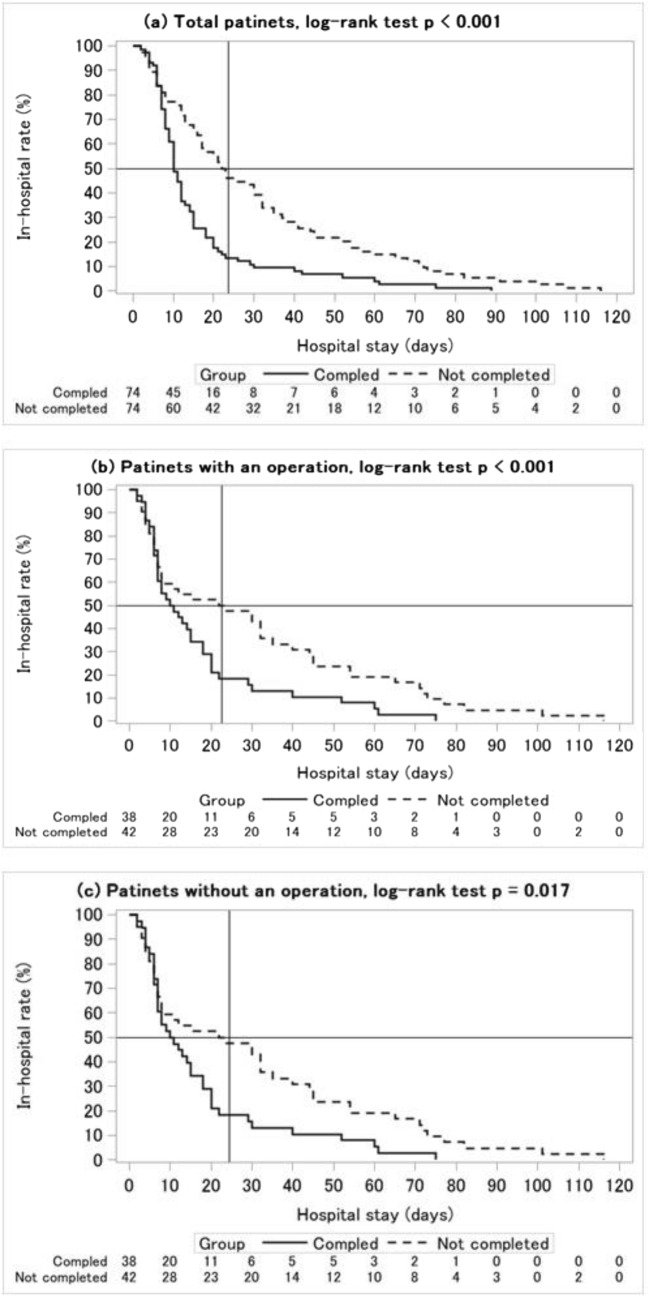
Discharge curve for (**a**) total patients, (**b**) patients without an operation, and (**c**) patients with an operation

All variables for data analysis were defined using items in the three raw databases (Fig. 1). The primary outcome of this study—length of hospital stay—was calculated as a difference between the date of hospital discharge and that of admission. CP completion, a basis of comparison between the groups, was defined as patients who have taken all the medical treatment registered on CP. Explorative variables for PSM were defined as follows: (1) sex; used as an item on the patient information database (Fig. 1), (2) age; difference between the date of hospital admission and birthday, (3) operation (yes or no); used as an the item on the patient information database (Fig. 1), (4) complication (yes or no); patients with complication were those with the main disease coded by the International Statistical Classification of Diseases and Related Health Problems 10th Revision and not registered on CPs, and (5) the second type of completion rate; defined as per the following equation:

$$ 1\hbox{-} \left[\mathrm{number}\ \mathrm{of}\ \mathrm{treatment}\ \mathrm{order}\ \mathrm{not}\ \mathrm{registered}\ \mathrm{on}\ \mathrm{CPs}\right]/\left[\mathrm{total}\ \mathrm{number}\ \mathrm{of}\ \mathrm{treatment}\ \mathrm{order}\right]. $$

### Statistical methods

For exclusion of bias using data items in EMRs, this study applied PSM methods to compare patients with CP completion and those without CP completion by 1-to-1 matching. Explorative variables for PSM were selected based on patient characteristics (Table 1, sex, age, operation, complication, and the second type of completion rate as explorative variable for PSM).

**Table 1:** Patient characteristics before propensity score matching

Variable	Number of patients (n)	CP completion (n)	Rate of CP Completion (%)
Total	227	152	67.0
Sex			
Male	139	90	64.7
Female	88	62	70.5
Age (years)			
Mean (SD)	66.8 (12.2)	66.6 (12.9)	–
Median (Min, Max)	68 (23, 86)	69 (23, 86)	–
0–54	31	23	74.2
55–59	18	11	61.1
60–64	28	15	53.6
65–69	47	32	68.1
70–74	39	28	71.8
>74	64	43	67.2
Operation			
No	105	62	59.0
Yes	122	90	73.8
Complication			
No	178	124	69.7
Yes	49	28	57.1
Completion rate (second type, %)			
Mean (SD)	24.25 (15.12)	18.71 (9.43)	–
Median (Min, Max)	21.0 (2.8, 81.7)	18.4 (2.8, 45.8)	–
0–10	34	32	74.2
>10–20	68	54	79.4
>20–30	67	47	70.1
>30–40	31	16	51.6
>40	27	3	11.1

*CP* Clinical pathway, *SD* Standard deviation

The main analysis was to compare length of hospital stay by completion of CPs, and existence of operation as follows: comparison of mean and standard deviation (SD) using the Student’s t test; estimation of the discharge curve based on the Kaplan–Meier’s method with the log-rank test. The result of the data analysis would visualize the causation between completion of clinical pathway and reduction in hospital stay, which was the theme of this study.

Furthermore, the additional analysis was to compare the frequency of deviated medical treatments that are described on CPs and not implemented. The Chi-square test was applied to compare the frequency in patients’ characteristics. The result of the data analysis would clarify the cause of the difference in the discharge curve by completion of CPs and existence of operation.

All data analyzes were performed using the SAS University Edition software (SAS Institute, US).

## Results

### Descriptive and outcome data

Tables 1 and 2 present the patient characteristics before and after PSM, respectively. The rate of CP completion rate (see Table 1) for females aged <55 and > 64 years old, those with an operation, those without complications, and those with ≤30% for the second type of completion rate were higher (67.0%) than that observed for total patients before application of PSM methods (*N* = 227). Because The completion rate decreased with the increase in the second type of completion rate, the second type of completion rate was not used to apply PSM.

**Table 2:** Patient characteristics after propensity score matching

Variable	With CP completion group (n)	Without CP completion group (n)
Total	74	74
Sex		
Male	37	48
Female	37	26
Age (years)		
Mean (SD)	61.1 (13.9)	67.0 (10.4)
Median (Min, Max)	64 (23, 84)	67 (29, 84)
0–54	20	8
55–59	8	7
60–64	12	13
65–69	16	15
70–74	6	11
>74	12	20
Operation		
No	38	42
Yes	36	32
Complication		
No	50	54
Yes	24	20

*SD* Standard deviation

### Main results: Discharge curve

Figure 2 shows six discharge curves by CP completion and the existence of an operation. The means and SDs of the hospital stay length were 23.6 ± 23.5 days (total patients), 24.5 ± 26.8 days (without an operation), and 22.6 ± 20.1 days (with an operation). The means and SDs of the hospital stay for the CP completed group vs the not completed group were 16.0 ± 16.2 days vs 31.3 ± 27.1 days (total patients, *p* < 0.001), 17.4 ± 17.8 days vs 31.0 ± 31.2 days (patients without an operation, *p* = 0.018), and 14.6 ± 14.4 days vs 31.6 ± 23.0 days (patients with an operation, p < 0.001). The *p* value of the log-rank test was <0.001 for total patients and patients with an operation. The p value was 0.017 for patients without an operation.

For a deeper interpretation of the discharge curve, there were two specific parameters we used, namely, the halving day (HD), which is the day at 50% of the in-hospital rate, and the rate of average stay (RAS), which is the in-hospital rate at the mean of the hospital stay. Horizontal and vertical reference lines are described in Fig. 2 to show both the HD and RAS. Although the value of the HD was about 10 days and the RAS was about 10% to 20% for the completion group, the values were about 22 to 24 days and 50% at the point of the intersection of the two reference lines of the discharge curve.

### Additional results: Deviated medical treatments on clinical pathways

Table 3 shows a list of the deviated medical treatments that were described on CPs but not implemented. It is remarkable that medical treatments involving intravenous drips occupied the first, second, and third rankings.

**Table 3:** Top ten largest number of medical treatments described on clinical pathway and not implemented

Ranking	Details	Frequency
1	Drip	388
2	Peripheral drip	161
3	Peripheral drip (side)	139
4	Local injection	112
5	Epidural anesthesia	112
6	Ropivacaine Hydrochloride (2 mg/mL)	94
7	Infusion (500 mL)	92
8	As-needed injection	90
9	Lactec D Injection (500 mL)	66
10	Internal medicine	56

Table 4 shows the frequency of patients in which the intravenous drip was not implemented (equal to the above first, second, and third rankings on Table 3) by the four variables on Table 1.

**Table 4:** Occurrence of intravenous drip unimplementation by patient characteristic

Variable		Number of patients	Unimplementation of drip		p value
			Number	Rate (%)	
Sex					
	Male	48	22	45.8	0.197
	Female	26	16	61.5	
Age (years)					
	0–54	8	4	50.0	0.080
	55–59	7	6	85.7	
	60–64	13	3	23.1	
	65–69	15	8	53.3	
	70–74	11	8	72.7	
	>74	20	9	45.0	
Operation					
	No	42	15	35.7	0.002
	Yes	32	23	71.9	
Complication					
	No	54	26	48.1	0.365
	Yes	20	12	60.0	

## Discussion

### Key results: Reduction in length of hospital stay

This study’s main finding was that the in-hospital rate for the CP completed group was less than that of the not completed group. In addition, the difference in the decrease in hospital stay length between the two groups was larger for patients who had operations. This result implies that a treatment plan with an operation contributes to improved efficiency in terms of a reduced hospital stay, as well as safety, with regard to the flowcharts associated with European Society for Medical Oncology guidelines [13–17]. This is because these flowcharts advise medical staff to perform an operation if possible at any stage (from first to fourth) and with any type (non-small cell or small cell) of lung cancer.

In addition, the two specific parameters (HD and RAS) showed an interesting trend in terms of hospital stay. The RAS value for the not completed group was equal to the mean of the hospital stay when the HD was 50%. This indicated that half the patients without a completed CP could be out of the hospital even if the medical staff stopped the medical treatments planned at the time of hospitalization. This may be due to an unforeseen complication; however, there may be other reasons, such as the hospital payment system. Recently, most hospitals with advanced equipment and staffing levels, including the University of Miyazaki Hospital, have implemented a payment system where the fee is decided by disease and treatment plan. This is termed the diagnosis procedure combination system [1]. Under this system, the fee decreases significantly if the length of the hospital stay passes the average. Therefore, patients without completed CPs may be out of the hospital as soon as possible to improve the hospital’s turnover rate.

### Additional results: Evaluation of incomplete clinical pathways

While the above section explained completed CPs’ effectiveness in relation to reduced hospital stay, this section discusses the issue of incomplete CPs. Because incomplete CPs occur with deviated medical treatments (when medical treatments registered on CPs are not implemented), it is important to clarify the difference between them to examine the frequency of the deviation. In this study, the medical treatment that was not implemented most often was the intravenous drip. While it is impossible to examine the details of the drip from EMRs, there are two kinds of intravenous drips: those involving an anticancer drug (equal to the assignment of chemotherapy) and those involving other drugs or treatments.

Historically, there have been three main methods for the treatment of cancer: operation, chemotherapy, and radiotherapy. In addition, various meta-analysis studies have evaluated the combination of these methods for typical types of lung cancer (e.g., second malignancies after radiotherapy for prostate cancer [18], chemotherapy in non-small cell lung cancer [19], radiotherapy plus epidermal growth factor receptor tyrosine kinase inhibitors [20], and other treatments [21–26]).

Therefore, subdividing the main treatment is important for examining the occurrence of deviated medical treatments on the basis of the difference of the rate of unimplemented intravenous drip whether an operation was conducted or not (*p* = 0.002, see Table 4). For patients who have received an operation or radiotherapy, comparing these treatments by the operation date or radiotherapy would be considered a main factor. For chemotherapy patients, unused anticancer drugs would be a main factor for consideration.

### Limitations

This study has several limitations. The first was the use of EMRs. Instead of using large-sized EMRs in this study, our data analysis was subjected to prefixed data items and standardized information content. For example, whether an intravenous drip was administered or not had a strong effect on whether the CPs were considered complete or incomplete. In addition, a cancer stage (from zeroth to fourth) would be useful to classify cancer patients. However, it is impossible to clarify and use them because of EMR standardization.

The second limitation was the study design. This was a retrospective and observational cohort study, as opposed to a prospective, interventional, and randomized trial. Thus, other potential confounders not recorded on EMRs were not considered. In addition, it is ethically impossible to assign patients to a complete or incomplete CP group at the time of hospitalization.

The third limitation was the lack of detailed CP information system for each medical treatment. Although several hospitals have developed an electronic CP system, only few have introduced a data warehouse that can classify medical treatments into CP registration or not. In other words, only few hospitals (including the University of Miyazaki Hospital) can decide CP completion using EMRs automatically. Despite the simple analytic method, it is difficult to apply other EMRs worldwide. Therefore, this limitation is more serious than other limitations and indicates the necessity of integrated data analysis described in the above section.

The fourth limitation was complex outcome in medicine as the third issue of the integrated analysis described in the above section. The present analytic method, especially the discharge curve, is specialized in evaluating the length of hospital stay. More concepts and methods for data analytics by satisfaction of multiple outcomes would be necessary to apply this method in real-time situations in medicine.

### Interpretation

The analytic method has high generalizability for evaluation of length of hospital stay, one of the most popular effectiveness outcomes using PSM and Kaplan–Meier’s curve with a little improvement of study evidence level. This improvement means that the design of this study, a retrospective cohort study using PSM matching, could be take a higher evidence level between typical prospective study (e.g., randomized clinical trial) and simple retrospective cohort study [27] because of exclusion of possibility of bias for data items in EMRs. Without regarding the third limitation, there would be high possibility to compare various outcomes (e.g., mortality rate, patient satisfaction).

Despite the significant results obtained herein by comparison of patients with CP completion and those without CP completion, however, the main interpretation from our key and additional results is the integrated analysis of medical treatments with or without registration of CPs based on the third study limitation. There are two factors associated with the integrated analysis as follows: (1) complex outcomes in medicine, (2) the best combination of medical treatments.

The first factor indicates that evaluation of various types of outcomes is crucial for medical data analysis. When developing a novel drug, satisfaction of safety outcomes and improvements in efficacy outcomes by regulation authorities (e.g., Food and Drug Administration in United States [28–30]) are necessary. To clearly demonstrate the impact of novel drugs, clinical trials for the development of such drugs must establish only one indicator as a primary endpoint based on the International Council for Harmonisation of Technical Requirements for Pharmaceuticals for Human Use. Therefore, these trials aim at improving statistical analysis to be able to include various factors in a single indicator (e.g., surrogate endpoint [31], composite endpoint [32]). Conversely, the type of data analysis demonstrated in the present study should satisfy several outcomes applicable to real-time situations in medicine. With an assumption of “N” number of outcomes required for satisfaction in data analysis, this approach could be defined as optimization of one outcome subject to “N – 1” number of other outcomes.

The second factor indicates the extraction of medical treatments to optimize N outcomes using data analytic techniques. When adverse events as considered a safety outcome and a service profit is considered as an efficacy outcome, it is possible to let the effectiveness be an indicator for optimization and the safety be a limiting condition, similar to firm’s economic theory (e.g., profit maximization and cost minimalization) [33]. A sufficient number of medical treatments would be necessary to decrease the adverse events. Conversely, expenditure on medical service would create a deficit if there are unnecessary tests and medication. For an advanced level, reinforcement learning would be useful to select medical treatments for optimization of various rewards as the complex outcome in medicine, similar to that in table games [34].

## Data Availability

Not Applicable.
